# Hiding in Plain Sight: Benefit of Abrasion and Laceration Swabs in Identification of Panton-Valentine Leucocidin (PVL)-Meticillin Resistant Staphylococcus aureus (MRSA) Colonisation in Military Personnel

**DOI:** 10.7759/cureus.39487

**Published:** 2023-05-25

**Authors:** Andy Matheson, Ross Hemingway, Marina Morgan

**Affiliations:** 1 Commando Training Centre Royal Marines, Ministry of Defence, Lympstone, GBR; 2 Health Centre, His Majesty's Prison (HMP) Leeds, Leeds, GBR; 3 Microbiology, Royal Devon and Exeter Hospital, Exeter, GBR

**Keywords:** meticillin resistant staphylococcus aureus, panton valentine leucocidin, screening, infection control, mrsa, pvl

## Abstract

Background

Outbreaks of Panton-Valentine Leucocidin (PVL)-producing methicillin-resistant *Staphylococcus aureus* (MRSA) skin and soft tissue infections (SSTIs) are a recurrent challenge for the Royal Marines at the Commando Training Centre (CTCRM). The intensity of commando training, its impact on skin integrity, and persistent colonisation reservoirs within the training centre have thwarted attempts to prevent these outbreaks.

Aim

To present an outbreak of PVL-producing MRSA SSTIs at a military training centre, demonstrating the benefit of additional abrasion and laceration swabs on the identification of colonised personnel and showing the effectiveness of a 10-day decolonisation regime.

Method

Following the identification of the outbreak of PVL-producing MRSA, all 36 members of the Recruit Troop underwent nasal MRSA screening to identify MRSA carriers. The screening was repeated on day 16 after completing an enhanced 10-day decolonisation regime. A third screening was conducted on the 110th day after a second peak of infection was identified. Various infection control measures, such as enhanced cleaning, restriction of movement and adjustments to the military training serials, were introduced to prevent further spread through the training centre.

Results

In this outbreak, two-thirds (eighteen) of the Recruit Troop suffered MRSA-PVL skin infections requiring antibiotic therapy and three required hospital admission for surgical management of their abscesses. The outbreak lasted 130 days, with two spikes in infections 10 weeks apart. The outbreak was successfully confined to one troop.

Conclusion

With concerns about low identification rates of carriers using nasal screening for MRSA, in this outbreak, we improved the identification of asymptomatic carriage with the simple step of additional culture swabs for all cuts and abrasions. Improved identification of colonised recruits, along with an enhanced decolonisation regime and rigid infection control practices, prevented the further spread of the clone through the training centre.

In a population with constant ongoing skin trauma, such as the military, contact sport athletes and iIV drug users, our results show that a culture of suitable abrasions/lacerations will improve the identification of MRSA colonisation compared with nasal swabs alone. Despite ongoing skin trauma and the logistical difficulties in delivering effective decolonisation during military training, decolonisation was successful in 79% of recruits after one decolonisation and 87% after the second 10-day decolonisation.

## Introduction

Skin and soft tissue infections (SSTIs) are a common and debilitating problem for the recruits at the Commando Training Centre Royal Marines (CTCRM). Whilst the majority of SSTIs are due to meticillin-sensitive *Staphylococcus aureus* (MSSA), there have been regular outbreaks of Panton-Valentine leukocidin (PVL)-producing MSSA and methicillin-resistant *S. aureu*s (MRSA) SSTI [1]. Resistance to first-line empirical antimicrobials combined with the impact of commando training on the recruits’ immune function (reduced sleep, variable nutrition, hard physical training) can lead to severe infections [2].

Additionally, colonised recruits at CTCRM are at high risk of spreading MRSA amongst their troop (single class of Royal Marine (RM) recruits, 30-60 in size with a shared living area) due to regular skin-to-skin contact with colleagues, crowded living conditions and informal sharing of items such as towels. A challenging consequence of commando training, and undoubtedly one of the most significant risk factors for skin infections at CTCRM, is the constant presence of cuts and abrasions on the skin of recruits (‘Woodbury rash’) [3].

*S. aureus* is carried by approximately one-third of the population, with around 20% being continually colonised, while other individuals are only intermittently colonised [2,4-6]. The challenge with intermittent colonisation is that it complicates outbreak control and testing. Patients might be mistakenly assumed to have been successfully decolonised when they are merely in a negative colonisation phase. This can result in an overlooked reservoir for the ongoing transmission of MRSA. Other challenges to outbreak control include the ‘sanctuary sites’ of the body, for example, the bowel [7,8], and the proclivity of certain strains to colonise skin rather than the more commonly tested sites, such as the nares or throat [4,6,8-10].

On average, 1300 recruits and 400 potential officers attend training courses at CTCRM annually. In this paper, we present the management of an outbreak of PVL-producing MRSA within an RM Recruit troop and the impact of swabbing broken skin in addition to the traditional nose and throat screening.

## Materials and methods

Two-thirds of the RM recruits in the troop suffered PVL-MRSA skin infections requiring antibiotic therapy. Each infected recruit missed an average of 17 days of training. Three of the troops required hospital admission for surgical management of their abscesses. The outbreak lasted 130 days over the autumn and winter of 2018/19, with two spikes in infection 10 weeks apart.

Identification of outbreak

Wound swabs, routine for all clinical presentations of SSTI, identified two recruits within a single troop with MRSA infection. Both isolates were resistant to gentamicin, ciprofloxacin, clindamycin, erythromycin, and trimethoprim and sensitive to rifampicin, mupirocin, linezolid and doxycycline. Isolates referred to the National Staphylococcal reference laboratory at Colindale, London, were confirmed to be Sequence Type (ST) ST22 CC 22, t309 and carried the PVL gene. ST 22 has recently been described as causing an outbreak within a surgical ward in a UK hospital setting [11]. There are several ST types currently circulating in the marine camp, almost all MSSA. This outbreak strain was an epidemic MRSA clone (E-MRSA-15) mainly reported in Europe, Australia and Canada, emerging with EMRSA-16 (ST36 CC30) in the 1990s in the UK [12]. The only oral options for single-agent oral therapy were doxycycline or linezolid. An outbreak was declared, and all recruits in the troop were brought to the Medical Centre for swabbing of their nose and any open wounds.

Control measures

An outbreak team from the Training Centre coordinated the introduction of infection control measures to prevent further spread. Items such as sleeping bags with a high fomite burden but damaged by high-temperature washes were exchanged for new ones. The troop remained housed in their multi-person dormitory when not on outdoor training, occupying the entire floor of an accommodation building. They did not share toilets or showers with any recruits from different troops or classes. At the declaration of an outbreak, the entire floor underwent a deep clean by an external cleaning company, wiping down all surfaces with chlorine-releasing disinfectant to remove dust and inactivate organisms, along with changing and disposing of laundry, bedding and curtains. An enhanced daily environmental cleaning protocol, including disinfectant product cleaning of all high-touch surfaces, was undertaken by the troops and supervised by appropriately trained military staff. The recruits were allowed increased access to the camp's launderette, which was given clear instructions on washing temperatures and handling of bedding and clothing. Enhanced hand hygiene measures were introduced, with monitored hand washing and surface cleaning at the shared canteen and gymnasium. Finally, since the normal commando training program involves repeated periods of exercising in remote training areas for days on end, their program was adapted to allow regular access to shower facilities to facilitate the triple therapy decolonisation.

Screening recruits for MRSA colonisation

Amies medium with charcoal nose and throat swabs were performed as a self-swab, supervised by nursing staff or Royal Navy/Royal Marine Medical Assistants on Days 1 and 16 (after first decolonisation) for all recruits. Further swabs of the nose/throat/wound were performed on day 110 after the medical team identified new clinical infections indicating a resurgence of the outbreak. Skin swabs were not performed on recruits with intact skin. MRSA screening cultures were performed using MRSA-selective agar (Biomerieux). Incubated as per standard operating procedures at 370 C, plates were inspected after 24 hours and 48 hours of incubation. Suspicious colonies were then characterised by matrix-assisted laser desorption ionisation-time of flight (MALDI-TOF) mass spectrometry (MS) (Biomerieux), and confirmation of PBP2A was confirmed by immunochromatographic assay (Alere PBP2A SA test). Screening for PVL-associated lukS-PV/lukF-PV genes was performed by real-time multiplex PCR, with molecular characterisation by spa typing undertaken by the national reference laboratory. 

Treatment of SSTI

Personnel with identified SSTI requiring antibiotics were treated as per the CTCRM Infection Policy with flucloxacillin and clindamycin or had case-specific advice from a Consultant Microbiologist. Repeated point-of-care CRP testing was performed on all patients with deranged clinical observations. Recruits requiring additional care and support, such as surgical drainage and IV antibiotics, were admitted to local secondary care.

Decolonisation regimen

All RM recruits undertook our local 10-day 'triple-agent' empirical decolonisation regimen repeated a month later. The 10-day regimen (mupirocin intra-nasal cream applied three times a day, daily chlorhexidine mouthwash, and daily shampoo/body wash with 4% chlorhexidine) was evolved in 2018 from the traditional five-day MRSA decolonisation hospital regimen for two reasons. Firstly, an increased failure rate has been noted, particularly with PVL-producing Staphylococcal strains in patients over the years. Secondly, the practical and logistical difficulties of effective decolonisation of troops undergoing training in the field were observed. All recruits received a verbal brief and demonstration from a member of the Nursing team and were given Patient Advice Leaflets on MRSA decolonisation.

## Results

The outbreak lasted 130 days, with peaks in infection two months apart. Thirteen recruits suffered infections requiring antibiotics during the first peak, with many developing a repeat infection in the second peak. Initial decolonisation success rates were higher than expected; however, the second infection peak indicates that recolonisation quickly developed (Table 1). 

**Table 1:** Baseline characteristics, impact on training and decolonisation rates.

Characteristics	
Outbreak duration (days)	130
Average age of recruits in troop (years)	23
Number of recruits in troop	34-36
Total number of recruits with infections	18 (average 1.8 infections per infected recruit)
Success rate of initial decolonisation	79%
Success rate of second decolonisation	87%

Most recruits will have small lacerations and wounds during their commando training at CTCRM. Higher rates of skin laceration and wounds were seen in recruits after returning from days spent in the outdoor training areas. Swabbing open wounds proved to be the most effective way to identify MRSA carriers (Table 2).

**Table 2:** MRSA swab results for the screening at Day 1, Day 16, Day 110. MRSA: Methicillin-resistant Staphylococcus aureus (MRSA).

	Day 1 - 1^st^ set swabs	Day 16 - 2^nd^ set swabs	Day 110 - 3^rd^ set swabs
Number of recruits in troop	34	35	36
Number of recruits with nasal swabs positive for MRSA	6	2	2
Number of recruits with open lacerations/abrasions	17	9	5
Number of recruits with laceration/abrasion swabs positive for MRSA	12	7	3
Total recruits with MRSA positive swabs	14/34 (40%)	8/35 (22%)	5/36 (19%)

Not all recruits had open wounds to swa. Fifty per cent (17/34) had wounds sampled initially, twenty-five per cent (9/35) had wounds when the second set of swabs was taken, and eleven per cent (5/42) had wounds swabbed at the third sampling. This outbreak highlights the practical difficulties in assessing and control of an outbreak since moving in and out of the troop by recruits over the 32 weeks was unavoidable since some recruits failed to progress through fitness and military tests or suffered injuries that required time out in rehabilitation, being "back-trooped". Three recruits that left commando training during the outbreak did not complete the full swab or decolonisation regime. Nine recruits joining the troop midway through the outbreak missed the first set of screening swabs, but none had positive swabs at any stage. Four recruits were transferred to other troops for further military training during the outbreak.

## Discussion

With a regular intake of new recruits from the UK and overseas, RM recruits are always at high risk of acquisition of new Spa types of S. aureus. Rapid and accurate identification of colonised recruits is essential to enable decolonisation, prevent further spread, and ensure the correct treatment of any SSTI. Previous studies of RM recruits at CTCRM demonstrated surprisingly low levels of nasal carriage of staphylococci despite high rates of concomitant SSTI [1].

Our results show that a culture of suitable abrasions/lacerations will improve the identification rate of those with MRSA colonisation over nasal swab culture.

There has been little change to the recommended wound care since the 1979 outbreak of Streptococcus pyogenes involving 1300 recruits. The outbreak was curtailed by education and encouraging troops to identify lesions, self-administer povidone-iodine spray to wounds and use medicated soap [3]. The 10-day decolonisation regimen was successful in 79% of recruits, much higher than the approximately 40% clearance often reported after five-day topical decolonisation [13]. Repeating two 10-day decolonisation regimens increased successful decolonisation to 87%. This improvement in successful decolonisation rate is similar to that achieved by five five-day decolonisation in a series of patients with skin abscesses due to PVL-producing staphylococci, where the number of symptomatic patients fell with each repeated decolonisation treatment and finally reached 89% following five decolonisations [14].

We believe the high success of MRSA decolonisation observed during our outbreak was due to three key factors, namely a very experienced RM training team's policing of the recruits' skin condition, a 10-day triple decolonisation protocol, and regular intensive patient education. A challenge of decolonisation in active military personnel is ongoing skin trauma during and after decolonisation and lack of showers when training in field conditions overnight. The RMs are investing in new clothing and equipment to reduce the impact of SSTI, which will allow for improved skin healing. Whilst smoking has been associated with a lower rate of staphylococcal colonisation [15], we saw no difference between smokers and non-smokers.

Limitations of our study include the lack of throat swabbing at the start of the outbreak, which likely contributed to an underestimate of initial MRSA carriage. Staphylococcal throat carriage is more common in our population age group and is reported in 20% of military recruits [10,16]. However, all troop recruits received decolonisation, including chlorhexidine mouthwash. Although the low sensitivity seen in the nose swabs was possibly due to the self-swabbing method used by the troops, others report no difference in sensitivity between nurse and self-swabbing tests [6]. Swabs of skin were not undertaken in recruits with no abrasions. In further studies, this must be introduced to provide a control group. The continued addition of new recruits to the troop during the outbreak has limited the ability to interrogate the results for transmission data, and numbers are very small, preventing statistical analysis. An important factor contributing to the high successful decolonisation rates was the impact of changing the training schedule to allow regular showering and application of topical disinfection with subsequent improvements in recruits' skin integrity. Ten days of decolonisation have been introduced at other UK military establishments that have reported significant reductions in PVL-staphylococcal infections (Morgan personal communication).

Looking ahead

Screening for MRSA using conventional culture and nasal swabbing alone overlooks some colonised patients, incidental carriers or hidden reservoirs [17]. We need to adopt newer technologies to identify carriers and those with overlooked reservoirs, such as the gut [8,18], potentially enabling control of outbreaks at an earlier stage. MRSA PCR screening quickly identified the carriage and targeting of antibiotic treatment in an ED study in the US [19]. PCR allows quicker and more accurate identification of MRSA colonisation and spread [20-22], together with mapping of transmission events. Metagenomics and microbiome evaluation of military trainees with SSTI would allow better detection of carriers [23] and identify those with S. aureus in a viable but non-culturable state [24]. Stool microbiome testing of those harbouring MRSA PVL in their gut will identify those reseeding skin with MRSA from the gut 'sanctuary site', already identified as a common reason for decolonisation failure [8]. Whole genome sequencing and outbreak analysis will provide more clarity into the unpredictable nature of colonisation in a population such as ours, allowing identification of the direction of the spread of the clones within the camp [21,25].

## Conclusions

In a population with ongoing skin trauma, such as the military or intravenous drug users (IVDU), our results show that a culture of suitable abrasions/lacerations will improve the identification of MRSA colonisation compared with nasal swabs alone. Moreover, it might be useful to further investigate repeated MRSA decolonisation regimens of longer duration.
